# PReS-FINAL-2081: Accelerometer-assessed daily activity in juvenile idiopathic arthritis

**DOI:** 10.1186/1546-0096-11-S2-P93

**Published:** 2013-12-05

**Authors:** M Noergaard, J Svensson, LB Andersen, T Herlin

**Affiliations:** 1 Department of Physiotherapy, Aarhus University Hospital, Skejby, Brendstrupgaardsvej 100, 8200 Aarhus N, Denmark; 2 Institute for Exercise and Sports Sciences, Copenhagen University, Copenhagen, Denmark; 3 Institute of Sports Science and Clinical Biomechanics, University of Southern Denmark, Odense, Denmark; 4 Department of Pediatrics, Aarhus University Hospital, Skejby, Brendstrupgaardsvej 100, 8200 Aarhus N, Denmark

## Introduction

Juvenile idiopathic arthritis (JIA) can be associated with decreased physical activity and impaired physical fitness. Within the past decade targeted therapy has led to improved disease control which has opened the possibilities for JIA-children to participate in dynamic sport activities. However, there are limited evidence-based recommendations as to the physical fitness needed to provide safe integration into sport activities for these children, and accurate, objective measures of physical activity are therefore needed.

## Objectives

To compare physical activity assessed by accelerometry in a cohort of children with JIA with normative data in gender- and age-related healthy children.

To relate levels of accelerometer-assessed physical activity to disease subcategory and activity, physical capacity and fitness, and pain.

## Methods

Seventy JIA-children aged 10 to16 years participated. Physical activity was assessed using the MTI ActiGraph accelerometer (Fort Walton Beach, FL, USA) worn on the hip with an elastic waist belt during the entire day for 6-7 days. Children failing to provide a minimum of 3 separate days of 8 hours of valid recording were excluded. Movement counts in a vertical plane were averaged over a period of time (epochs), here using 10 sec. Disease parameters (subcategory, ESR, JADAS10/-27), physical tests (6-minute-walk test (6MWT), indirect Watt-max test (WmT)) and a one-week pain diary using Faces Pain Scale-Revised (FPS-R) were also obtained.

## Results

Valid data were obtained from 61 JIA-children (10 systemic, 10 RF-neg, 6 RF-pos, 18 persistent oligo, 16 extended oligo, 4 psoriatic and 2 enthesitis related JIA). For each patient data from 236 ± 93 healthy age-related controls were compared. We found significantly reduced mean count/min in JIA-children (457 ± 172) compared to healthy age-related controls (518 ± 84, p = 0.004). Accelerometer counts > 1000/min reflecting low activity (e.g. walking) was registered 97.1 ± 40.4 minutes compared to 117.2 ± 23.5 in controls (p < 0.001) and counts >2500/min reflecting highly dynamic activity (e.g. running) was registered in 31.3 ± 19.4 min. compared to 38.6 ± 7.7 min. in controls (p = 0.002). Lowest activity was observed in the RF-pos JIA and highest in the persistent oligoarticular and psoriatic JIA subcategory. The accelerometry data were negatively correlated to ESR, JADAS10/JADAS27, 6MWT distance, and WmT but not to pain (one-week FPS-R diary).

## Conclusion

Though disease activity is better controlled and JIA-children are less physically impaired nowadays, they are still significantly less physically active than age- and gender-comparable healthy controls both regarding low and high physical intensity, but dependent on disease subcategory. Surprisingly, self-reported pain did not seem to have a significant impact on physical activity.

## Disclosure of interest

None declared.

